# Supplementary material for: ARMH3 acts as a central scaffold at the Golgi/TGN through interactions with Arl5, GBF1, and PI4KB
Source: J Biol Chem. 2026 Jun 29;302(8):113307. doi: 10.1016/j.jbc.2026.113307 (PMC13427656; doi:10.1016/j.jbc.2026.113307)
Supplement: Supporting Figures and Tables [file mmc1.pdf]

Supplementary Information

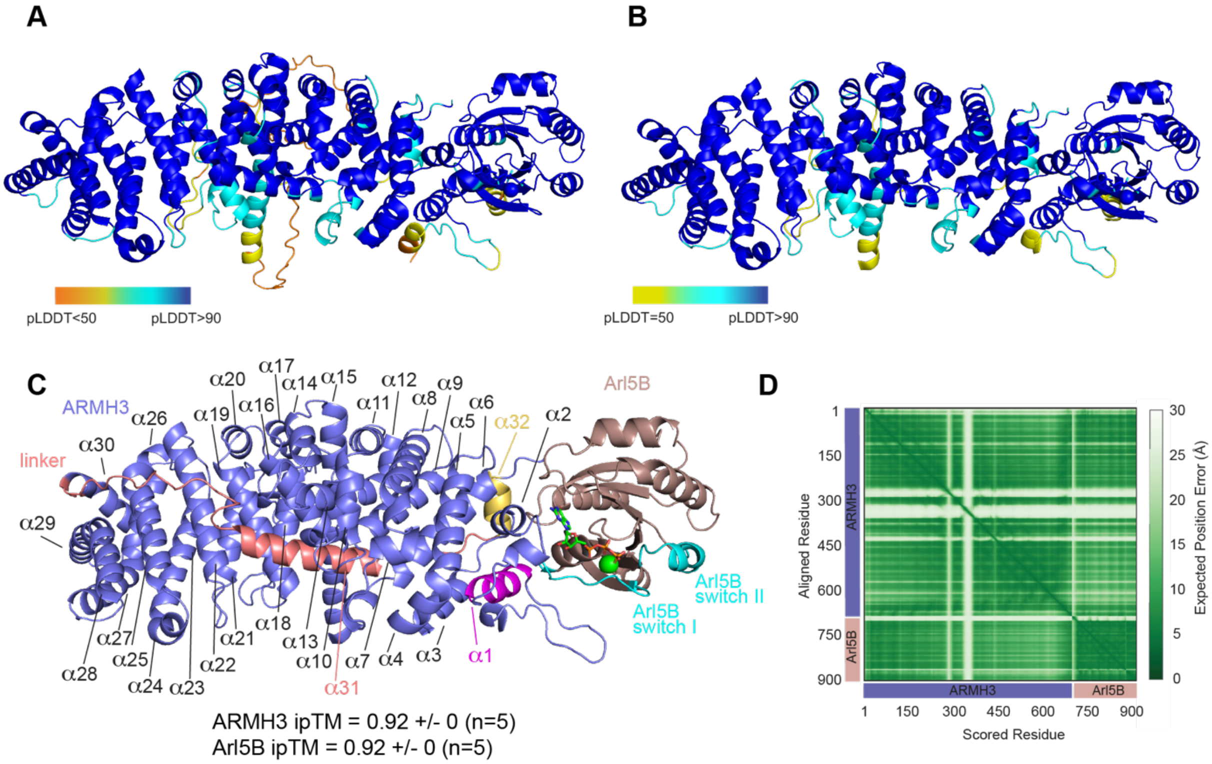

Figure S1: AlphaFold3 modeling of the ARMH3-Arl5B complex.

A) Best model (seed 2, n=5) of the Arl5B-ARMH3 AlphaFold3 prediction with cofactors GTP and Mg^{2+} coloured by pLDDT. chain_pair_iptm and chain_pair_pae_min information for individual seeds in source data.

B) The same model coloured by pLDDT, with regions of low confidence removed.

C) Model shown in panel B, coloured by chain. Helices in ARMH3 are numbered and labelled. ipTM scores represent the average across all five models in the top seed, with error shown as standard deviation.

D) Predicted aligned error (pae) plot for seed 2 of Arl5B in complex with ARMH3 and cofactors GTP and Mg^{2+}.

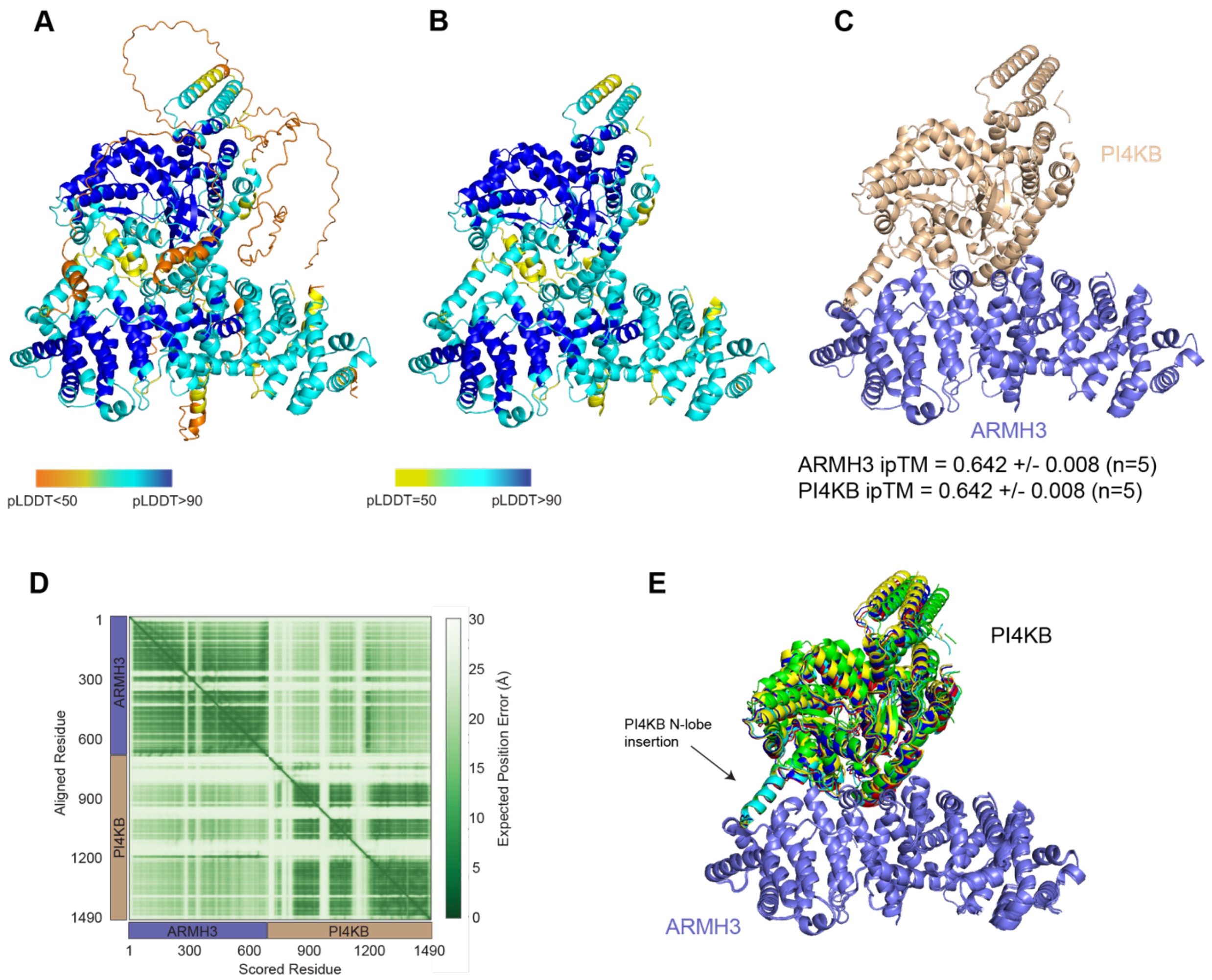

Figure S2: AlphaFold3 modeling of the PI4KB-ARMH3 complex.

A) Best model (seed 5, n=5) of the ARMH3-PI4KB AlphaFold3 prediction coloured by pLDDT. chain_pair_iptm and chain_pair_pae_min information for individual seeds in source data. **B)** The same model coloured by pLDDT, with regions of low confidence removed.

C) Model shown in panel B, coloured by chain. ipTM scores represent the average across all five models in the top seed, with error shown as standard deviation.

D) Predicted aligned error (pae) plot for seed 5 of PI4KB in complex with ARMH3.

E) Alignment of all five seeds onto ARMH3; PI4KB N-lobe insertion indicated with arrow.

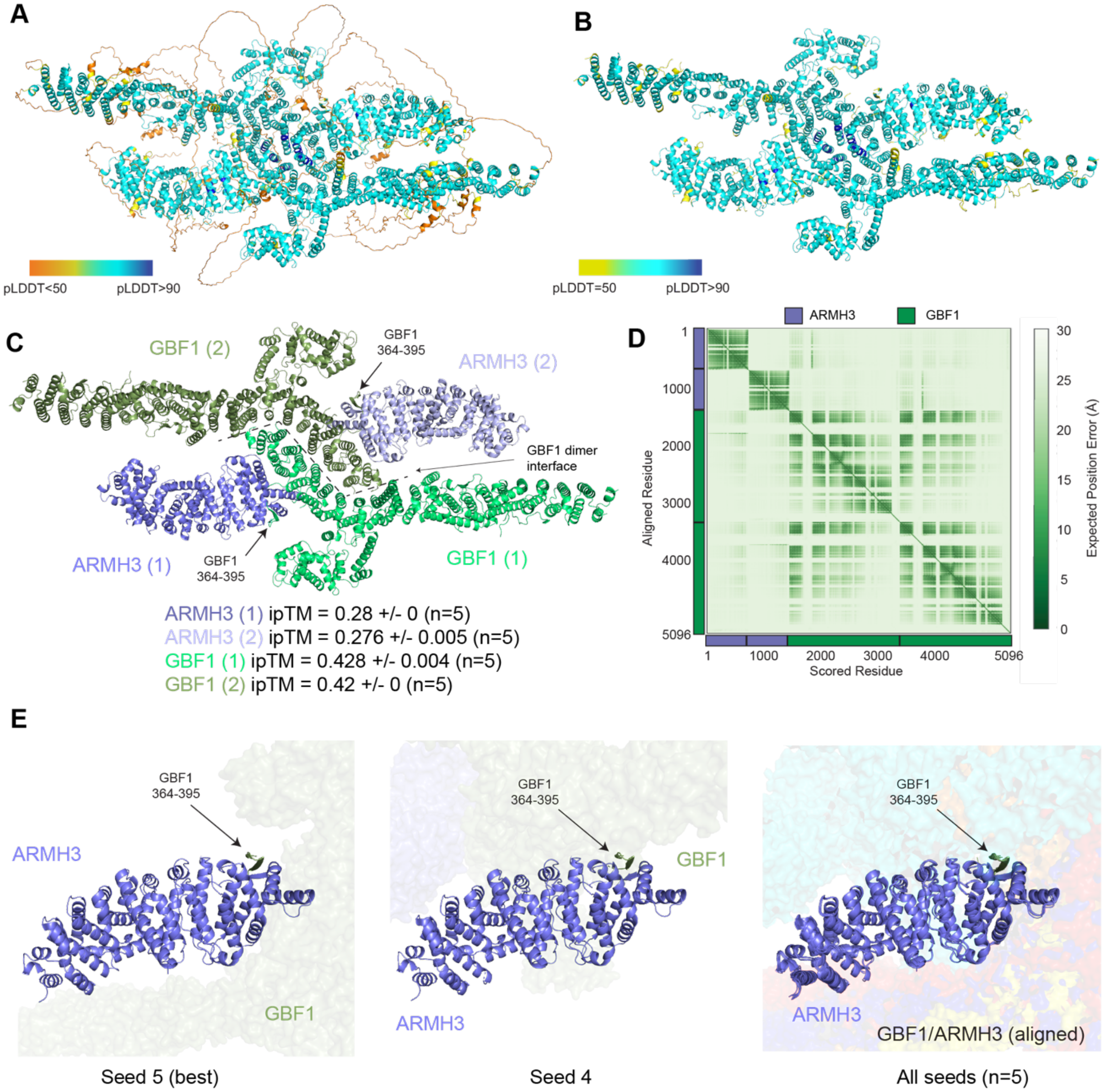

Figure S3: AlphaFold3 modeling of the ARMH3-GBF1 complex.

A) Best model (seed 5, n=5) of the 2xARMH3-2xGBF1 AlphaFold3 prediction coloured by pLDDT. chain_pair_iptm and chain_pair_pae_min information for individual seeds in source data.

B) The same model coloured by pLDDT, with regions of low confidence removed.

C) Model shown in panel B, coloured by chain. Predicted GBF1 dimer interface is indicated by the dashed line, arrows indicate GBF1 residues 364-395. ipTM scores

represent the average across all five models in the top seed, with error shown as standard deviation.

D) Predicted aligned error (pae) plot for seed 5 of 2xGBF1 with 2xARMH3.

E) Zoom-ins showing predictions of ARMH3 in complex with full-length GBF1, with one ARMH3 and GBF1 residues 364-395 shown as cartoons and the remainder of the model shown as a surface. Left and middle panels show select predictions of the GBF1-ARMH3 interface, while the right panel shows all five predictions aligned onto ARMH3. GBF1 residues are indicated with an arrow.

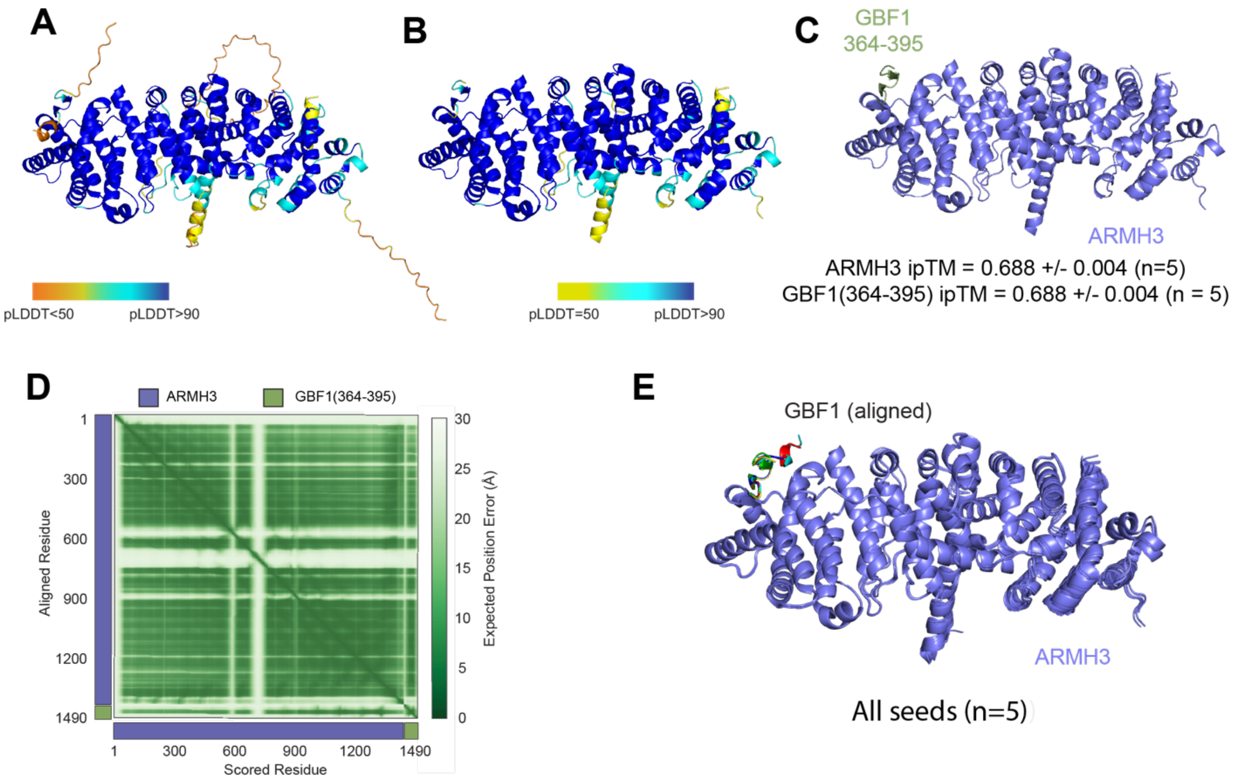

Figure S4: AlphaFold3 modeling of the ARMH3-GBF1(364-395) complex.

A) Best model (seed 4, n=5) of the ARMH3-GBF1(364-395) AlphaFold3 prediction coloured by pLDDT. chain_pair_iptm and chain_pair_pae_min information for individual seeds in source data.

B) The same model coloured by pLDDT, with regions of low confidence removed.

C) Model shown in panel B, coloured by chain. ipTM scores represent the average across all five models in the top seed, with error shown as standard deviation.

D) Predicted aligned error (pae) plot for seed 4 of GBF1(364-395) in complex with ARMH3.

E) Alignment of all five seeds onto ARMH3.

Table S1. Binding constants for the different ARMH3 complexes.

Immobilised Ligand	Analyte	K_D (nM)	SD (nM)	Kon (1/Ms)	SD	Koff(1/s)	SD	Assoc. X2
**Arl5B (GTPγS)**	ARMH3	75.4	18	1.74E+05	7.59E+05	1.22E-02	2.99E-03	0.4944
**PI4KB isoform 2**	ARMH3	6.2	1.0	8.35E+05	3.55E+04	5.12E-03	6.76E-04	0.1425
**GBF1 (364-395)**	ARMH3	320	80	1.33E+05	2.76E+04	4.08E-02	2.44E-03	0.5545

Table S2. AlphaFold3 confidence metrics for ARMH3 complex predictions. Values are averages plus/minus standard deviation for all five models in top seed.

**ARMH3-Arl5B(GTP) – seed 2**				
	iptm	ptm	chain_pair_iptm	
ARMH3	0.92 ± 0	0.84 ± 0	ARMH3-Arl5B	0.87 ± 0
Arl5B	0.92 ± 0	0.85 ± 0	Arl5B-GTP	0.94 ± 0
GTP	0.8 ± 0.007	0.668 ± 0.004	Arl5B-Mg2+	0.94 ± 0
Mg2+	0.794 ± 0.005	null		
**ARMH3-PI4KB – seed 5**				
	iptm	ptm	chain_pair_iptm	
ARMH3	0.642 ± 0.008	0.78 ± 0	ARMH3-PI4KB	0.642 ± 0.08
PI4KB	0.642 ± 0.008	0.66 ± 0		
**ARMH3(x2)-GBF1 dimer – seed 5**				
	iptm	ptm	chain_pair_iptm	
ARMH3 (1)	0.28 ± 0	0.74 ± 0	ARMH3 (1)-GBF1 (1)	0.488 ± 0.004
ARMH3 (2)	0.276 ± 0.005	0.74 ± 0	ARMH3 (2)-GBF1 (2)	0.476 ± 0.005
GBF1 (1)	0.428 ± 0.004	0.558 ± 0.004	GBF1 (1) - GBF1 (2)	0.568 ± 0.004
GBF1 (2)	0.42 ± 0	0.556 ± 0.005		
**ARMH3-GBF1(364-395) – seed 4**				
	iptm	ptm	chain_pair_iptm	
ARMH3	0.688 ± 0.004	0.83 ± 0	ARMH3-GBF1(364-395)	0.688 ± 0.004
GBF1(364-395)	0.688 ± 0.004	0.27 ± 0		
**ARMH3-Arl5B-PI4KB(481-516) – seed 5**				
	iptm	ptm	chain_pair_iptm	
ARMH3	0.87 ± 0	0.844 ± 0.005	ARMH3-Arl5B	0.874 ± 0.005
Arl5B	0.758 ± 0.004	0.846 ± 0.005	ARMH3-PI4KB(481-516)	0.702 ± 0.004
PI4KB(481-516)	0.29 ± 0.007	0.302 ± 0.004	Arl5B-GTP	0.946 ± 0.005
GTP	0.636 ± 0.005	0.67 ± 0	Arl5B-Mg2+	0.94 ± 0
Mg2+	0.61 ± 0	null		
**ARMH3-Arl5B-GBF1(364-395) – seed 1**				
	iptm	ptm	chain_pair_iptm	
ARMH3	0.86 ± 0	0.85 ± 0	ARMH3-Arl5B	0.88 ± 0
Arl5B	0.75 ± 0	0.848 ± 0.004	ARMH3-GBF1(364-395)	0.676 ± 0.005
GBF1(364-395)	0.266 ± 0.005	0.238 ± 0.004	Arl5B-GTP	0.94 ± 0
GTP	0.626 ± 0.005	0.666 ± 0.005	Arl5B-Mg2+	0.94 ± 0
Mg2+	0.606 ± 0.005	null		

Table S3. Key reagents/resources.

REAGENT or RESOURCE	SOURCE	IDENTIFIER
**Bacteria and Virus Strains**		
E. coli XL10-GOLD KanR Ultracompetent Cells	Agilent	200317
E. coli DH10EMBacY Competent Cells	Geneva Biotech	DH10EMBacY
C41 (DE3) chemically competent cells	Lab Stock	
**Chemicals, peptides, and recombinant proteins**		
Deuterium Oxide 99.9%	Sigma-Aldrich	151882-10X1ML
ATP	Sigma-Aldrich	A7699-1g
GTPγS Tetralithium salt	Millipore Sigma	10220647001
$MgCl_2$	Caledon Laboratory Chemicals	4720-01-01
Ethylenediaminetetraacetic Acid (EDTA), Electrophoresis Grade	ThermoFisher Scientific	ICN800682
Phosphatase, Alkaline-Agarose from Calf Intestine	Sigma-Aldrich	P0762
**Deposited Data**		
Mass spectrometry proteomics data - PI4KB-ARMH3	https://www.ebi.ac.uk/pride/	PXD076708
Mass spectrometry proteomics data - Arl5B-ARMH3, GBF1(1-709)-ARMH3, GBF1(364-395)-ARMH3	https://www.ebi.ac.uk/pride/	PXD080671
**Recombinant DNA**		
Arl5A (15-179)	This paper	MS161
Arl5B (15-179)	This paper	MS133
ARMH3 WT - *Sf9*	(12)	JM75
ARMH3 WT - *E. coli*	(12)	JM115
ARMH3 K53E	(12)	MS127
ARMH3 K24E	This paper	MS166
ARMH3 H93E	This paper	GK6
Arl5B (15-179) E163K	This paper	GK5
PI4KB WT - *Sf9*	(12)	SS196
PI4KB WT - *E. coli*	(12)	JM34

PI4KB RL494EA	(12)	JM109
ARMH3 D549R	This paper	MS128
GBF1(1-709)	This paper	MS165
GBF1(364-395)	This paper	MS124
GBF1(364-395) R381E	This paper	MS190
GBF1(364-395) Y377E	(21)	MS149
**Software and algorithms**		
PDBePISA	EMBL-EBI	https://www.ebi.ac.uk/pdbe/pisa/
AlphaFold3	Google Deepmind	https://alphafoldserver.com/
HDExaminer	Trajan Scientific and Medical	http://massspec.com/hdexaminer
Thermo Xcalibur Data Acquisition and Interpretation Software	ThermoFisher Scientific	https://www.thermofisher.com/
GraphPad Prism 7	GraphPad	https://www.graphpad.com/
FragPipe (v18.0, v23.1)	Nesvizhskii Lab – University of Michigan	http://fragpipe.nesvilab.org/
Adobe Illustrator 2026	Adobe	https://www.adobe.com/products/illustrator.html
EsPript 3.0	SBGrid consortium	https://espript.ibcp.fr/
PyMOL	Schroedinger	http://pymol.org/
**Other**		
Sf9 insect cells for expression	Expression Systems	94-001S
Octet HIS1K Biosensors	Sartorius	18-5120
Regen Buffer (Salt, 50mL)	GatorBio	120012
Anti-His (HIS) Probes	GatorBio	160009
Gator BLI 96-Tilt Plate	GatorBio	130162
Affipro Protein Pepsin Column, 2.1 mm X 20 mm, 69.3 µL	Affipro	AP-PC-001
ACQUITY UPLC BEH C18 1.7 µm, 2.1 mm x 5 mm	Waters	186004629
ACQUITY UPLC Peptide BEH C18 Column, 300Å, 1.7 µm, 100 mm X 2.1 mm	Waters	186003686

84

85
